# Nanoemulsion-Based Multilayer Films for Ground Beef Preservation: Antimicrobial Activity and Physicochemical Properties

**DOI:** 10.3390/molecules28114274

**Published:** 2023-05-23

**Authors:** Fatemeh Baghi, Sami Ghnimi, Emilie Dumas, Nour-Eddine Chihib, Adem Gharsallaoui

**Affiliations:** 1 University of Lyon, Université Claude Bernard Lyon 1, CNRS, LAGEPP UMR 5007, F-69622 Villeurbanne, Francesghnimi@isara.fr (S.G.);; 2 Higher Institute of Agriculture and Agri-Food Rhone-Alpes, ISARA, 23 Rue Jean Baldassini, F-69007 Lyon, France; 3 University of Lille, CNRS, INRAE, Centrale Lille, UMR 8207, UMET—Unité Matériaux et Transformations, F-59000 Lille, France; nour-eddine.chihib@univ-lille.fr

**Keywords:** pectin film, nanoemulsion, ethylcellulose, antimicrobial activity, multilayer packaging

## Abstract

This study aimed to improve the physical, mechanical, and biological properties of a monolayer pectin (P) film containing nanoemulsified *trans*-Cinnamaldehyde (TC) by incorporating it between inner and outer layers of ethylcellulose (EC). The nanoemulsion had an average size of 103.93 nm and a zeta potential of −46 mV. The addition of the nanoemulsion increased the opacity of the film, reduced its moisture absorption capacity, and improved its antimicrobial activity. However, the tensile strength and elongation at break of the pectin films decreased after the incorporation of nanoemulsions. Multilayer films (EC/P/EC) showed a higher resistance to breaking and better extensibility compared to monolayer films. The antimicrobial activity of both mono and multilayer films was effective in inhibiting the growth of foodborne bacteria during storage of ground beef patties at 8 °C for 10 days. This study suggests that biodegradable antimicrobial multilayer packaging films can be effectively designed and applied in the food packaging industry.

## 1. Introduction

Plastic packaging represents the largest use of plastics in the market and allocates nearly half of the plastic waste produced globally [1,2]. Due to the big concern of plastic pollution, biodegradable materials have been gaining importance to replace conventional plastic packaging. Biopolymers from plant, animal, or microbial sources such as polysaccharides, proteins, and lipids are promising natural materials for producing biodegradable packaging [3]. Biopolymers show interesting film-forming properties, although some barrier and mechanical properties need to be improved to meet the needs of packaging applications. Pectin is a polysaccharide commonly used in food industries. It is a structurally heterogeneous polysaccharide found in the primary cell walls and middle lamella of higher plants. It has a good gel-forming property and could be a matrix for the production of biodegradable, safe, and edible packaging films [4,5]. Pectin has several food applications, such as gelling agent, thickening agent, and stabilizer. It is also used in the medical and pharmaceutical industries for biomedical applications, including drug delivery, tissue engineering, and wound healing [6]. Pectin is a typical linear heteropolysaccharide formed by D-galacturonic acid linking with α-1, 4 glycosidic bonds. Pectin has a chemical structure with many negatively charged carboxyl groups, and it easily interacts with metal cations or active molecules with cationic groups [7].

On the other hand, a new trend is emerging of health-conscious consumers expecting fresh food products without synthetic preservatives. In this regard, natural bioactive molecules with antimicrobial activity can be used for extending the shelf life of food. Natural antimicrobial agents have different origins, such as plants, animals, or microbial metabolisms [8]. These natural antimicrobial agents could be incorporated in the thin layer of packaging films, thus forming active packaging materials. In the active packaging films, the biomolecules will be released to the surface of perishable foods to maintain their quality and extend their shelf life. Many biomolecules not only act as antimicrobials, but also have antioxidant activity [9].

Essential oils (EOs) are considered as natural antimicrobial compounds; they are aromatic and oily liquids obtained from different parts of plants such as flowers, buds, seeds, leaves, twigs, bark, herbs, wood, fruits, and roots [10]. The *trans*-Cinnamaldehyde (TC) extracted from cinnamon (60–75% of the total oil) is a well-known natural antimicrobial agent, effective against a broad spectrum of pathogenic microorganisms [11]. Although essential oils have some disadvantages, such as volatility, unfavorable taste, and odor, these can be resolved by encapsulating them. Encapsulation is the entrapment of active ingredients within a wall material to protect them against deterioration, volatile losses, or premature interactions with other ingredients, which could induce long-term releasing [12,13]. Moreover, encapsulation in nanosized particles could be more effective than in microsized particles by providing a higher active surface and efficiency of EOs to destroy the cell wall of the target microorganisms [14].

The production of pectin-based active films has been investigated due to its ability to carry biomolecules in addition to the other interesting properties of pectin mentioned before [7]. Nisar et al. [15] studied citrus pectin films integrated with clove bud essential oil. Their results showed an enhancement of the water barrier properties of the active films, a better film opacity, more flexibility and resistance to breakage, a slightly higher degradation temperature, as well as an antimicrobial activity against three common foodborne bacteria. Furthermore, pectin films activated by marjoram essential oil were assessed by Almasi et al. [16]. The physical characterization tests demonstrated the compatibility between pectin and loaded nanocarriers. Alvarez et al. [17] demonstrated the antimicrobial efficiency of oregano essential oil-pectin edible films against *Escherichia coli* O157:H7, *Salmonella choleraesuis*, *Staphylococcus aureus,* and *Listeria monocytogenes*. In the same study, the results exhibited the effective reduction in total coliforms, yeasts, and molds in shrimp and cucumber slices stored at 4 °C for 15 days. In another study, nanoemulsions containing oregano, thyme, lemongrass or mandarin essential oils were incorporated into a high methoxyl pectin matrix to elaborate active films [18]. Antimicrobial activity against *Escherichia coli* and *Listeria innocua* was obtained, but it decreased significantly during storage regardless of the EO type, which was related to the loss of volatile compounds over time.

Nonetheless, the film-forming potential of pectin has to be improved to meet the needs for food packaging applications, such as appropriate mechanical and barrier properties. More importantly, its hydrophobicity should also be modified because it can degrade easily when in contact with wet-packed food. In this regard, the use of hydrophobic biopolymers can deal with this difficulty. Among various types of biopolymers, ethyl cellulose (EC) is another polysaccharide made by the chemical substitution of the naturally occurring polymer, cellulose, and it has excellent film-forming properties—especially after adding plasticizers. EC is a water-insoluble cellulose ether with favorable mechanical properties, is non-toxic, and has a relatively low cost [19].

Slowing the release rate of active compounds for their prolonged activity is a considerable point to design an active packaging system [20]. Generally, it is rather difficult to find one biopolymer that has all the demanded properties. In this regard, packaging containing layers of different materials could provide desirable properties, such as higher barrier and mechanical strength or heat sealability, which no single material possesses. Furthermore, this type of packaging material has also been proposed to control the release of active compounds from the packaging toward the packed food [21]. Multilayer active films are composed of three layers: barrier layer with high barrier properties preventing the loss of active substances to the environment, matrix layer containing the active substance and showing very fast diffusion, and control layer with lower swelling ability than matrix layer, controlling the release of the active agent to the food. Thickness, chemical composition, and diffusivity of the control layer are important factors which should be considered in designing the multilayer film for each type of food [21,22].

Active multilayer packaging was designed with different biopolymers. For example, zein-based mono and multilayer films were loaded with spelt bran and thymol (35% *w/w*) [23]. The authors developed various composite systems to evaluate the effect of the thickness of the layers and the amount of biodegradable fibers on thymol release rate. Their results demonstrated a decrease in thymol release rate with the increase in film thickness, and conversely, a significant increase in thymol release rate with the increase in bran concentration for both mono and multilayer films. The release of vanillin, as a natural antimicrobial ingredient, from poly vinyl alcohol (PVA) and bacterial cellulose mono and multilayer films was also investigated [24]. A multilayer film structure of two external control layers and an inner active layer were developed to obtain functional food packaging materials. Multilayer film consists of three-layer films: two external bacterial cellulose membranes and an inner layer containing vanillin. The inner layer was prepared by adding vanillin at different concentrations (0.1% and 0.5% *w/w*) directly in PVA–cellulose dispersion. As a result, it was proved that vanillin release was influenced by film composition. In the case of multilayered films, the release rates were lower, indicating a slower diffusion process with promising potential to achieve a controlled release of vanillin for extending the shelf life of food. In another work, the controlled release of lysozyme from poly vinyl alcohol film (PVOH) was studied. Results suggest the possibility of controlling the rate at which lysozyme is released from the PVOH film, and an extended antimicrobial activity has been observed [25]. Moreover, sorbic acid as an antimicrobial agent was used for making active multilayer films from poly(vinyl) alcohol (PVA) and bacterial cellulose. In fact, combining bacterial cellulose and PVA presents many advantages, such as higher antimicrobial activity together with odorless, nontoxicity, chemical resistance and good mechanical properties. However, high moisture sensitivity was considered a drawback in the use of both bacterial cellulose and PVA, which limits the applications of these films in food packaging [26].

In the present work, we aim to develop multilayer films, including two outer layers of EC, to isolate an inner active layer of pectin incorporated with nanoemulsions (PNE: Pectin/Nano-Emulsion) of *trans*-Cinnamaldehyde as an antimicrobial agent. The contribution of this study lies in the improvement of pectin film properties by the multilayer technique. This study investigated the mechanical properties of these multilayer films and their antimicrobial activity against pathogenic microorganisms, as well as their efficiency to extend the shelf life of packed ground beef meat.

## 2. Results and Discussion

### 2.1. Particle Size and Homogeneity of Nanoemulsions

Many studies revealed the effect of the size of nanoemulsions on their biological activities. Xiong et al. [27] highlighted that loading of oregano essential oil and resveratrol nanoemulsions on pectin improved the meat preservation which has been attributed to their smaller particle size with enhanced preservative function and stability. Therefore, two steps of high-speed and high-pressure homogenization were employed in this study to reduce the size to around 100 nm. It is predicted that the nanosized particles of the antimicrobial agent could penetrate the cell wall of microorganisms and destroy them [28]. The average size of nanoparticles of TC was 103.93 ± 2.17 nm. The polydispersity index (PDI) is a means of investigating the homogeneity of nanoemulsions. In general, PDI values < 0.05 are more common to monodisperse samples, while values > 0.7 are considered to be a broad size (e.g., polydisperse) distribution of particles (ISO standards ISO 22412:2017 and ISO 22412:2017) [29]. In this study, the measured polydispersity index (PDI) of TC nanoemulsions was 0.24 ± 0.01, which is an indicator of monodispersity.

### 2.2. Zeta Potential

Zeta potential is the potential at the slipping plane between the particle and associated double layer with the surrounding solvent. At a large negative or positive zeta potential (generally taken as +30 and −30 mV), particles tend to repel each other and avoid coming together and flocculating [30]. The average zeta potential measured for nanoemulsions of TC was 46 ± 2 mV, which indicated a good stability of the TC nanoemulsions.

### 2.3. Thickness of Films

Thickness has an impact on the mechanical, barrier, and biological properties of active films [31]. The thickness of all samples is shown in Table 1. The thickness measurement showed an increase of about 40% for PNE films compared to pectin films, which can be attributed to the incorporation of nanoemulsions of TC into the matrix of pectin and the increase of dry matter in the same volume of film-forming solution cast into the petri dishes. The monolayer of EC had a thickness of 105.33 μm ± 7.02, which is about two times thicker than the monolayer of pectin. This difference is mainly due to the percentage of pectin and ethyl cellulose in film-forming solutions, which are 6% and 3%, respectively. Additionally, the percentage of glycerol, 2% for ethyl cellulose solution and 1% for pectin solution, could have an effect on this parameter. As expected, multilayer film samples were thicker, and the thickness was about 285.67± 18.65 μm—a combination of the thicknesses of the two layers of EC and the middle layer of PNE.

### 2.4. Opacity of Films

Transparency of film packaging is an important index in terms of overall appearance and consumer acceptance [32]. Lower opacity value indicated more transparency. The opacity of the different films is shown in Table 1. Pectin films without the incorporation of NE appeared clearer and transparent. The opacity of the pectin film markedly increased when TC nanoemulsions (ratio P:NE = 1:1) were added, resulting in less transparency for the active film (PNE). This considerable change could be ascribed to the coalescence, light-scattering, and creaming effect generated by the distribution of oil particles, droplets of nanoemulsion of TC (NE), during the drying process of the films, which promoted the surface coarseness of the film samples due to the presence of large TC droplets [33]. The reduction in transparency of pectin films by adding essential oils has also been reported by Nisar et al. [15], who made citrus pectin films integrated with clove bud essential oil, resulting in more opaque films by increasing the essential oil concentration up to 1.5%. The transparency of EC films was in agreement with that reported by Yang et al. [19]. Multilayer films showed an opacity value of 2.02, which could be due to the isolation of the PNE layer by two layers of EC that are rather transparent with an opacity of 0.97 (Table 1). Indeed, this transparency induced by EC could be considered as one of the advantages of the multilayer technique applied for improving the properties of the active films of pectin.

### 2.5. Moisture Absorption

The moisture absorptions of the different films are shown in Table 1. Pectin films have the highest capacity to absorb humidity which is around 22.54%. This moisture absorption capacity of pectin films decreased to 9.40% by incorporating the nanoemulsions of TC. The same reduction in the moisture absorption percentage of pectin films by adding nanoemulsions of marjoram essential oil was reported by Almasi et al. [16]. This reduction in moisture could have an effect on the water barrier properties. In general, better water barrier properties of pectin-based films are compatible with less moisture absorption. This is mainly due to the reduction in the diffusion of water molecules caused by adding nanoemulsion of essential oils which increases the tortuosity path of the polymer chains [34]. The moisture absorption of the monolayer of EC was around 7.21%. It was significantly less than that of the pectin sample because of its hydrophobic nature. However, the moisture absorption of multilayer films was about 15.35%. This increase could be explained by the method applied in this test. Indeed, the samples were cut, and they were placed in direct contact with the humidity of the air in an environment at 75% RH (NaCl saturated solution) for a considerable time during which water molecules could penetrate between the layers and be absorbed by the middle layer of PNE as well as the two layers of EC on the two sides of the surface. Therefore, moisture absorption increased for multilayer films compared with each monolayer separately.

### 2.6. Mechanical Properties

TS is considered as the maximum stress supported by the film before breaking, which presents stretching capacity. EB is a mechanical property that provides information about the ability of a material to undergo significant deformation before failure, and it indicates film resistance to break. YM represents film stiffness, and the stiffer material will have a higher elastic modulus. These mechanical properties were measured to investigate the effect of the incorporation of TC nanoemulsion in the pectin film, as well as the impact of the multilayer technique. The results are summarized in Table 2. Compared with pectin films, the incorporation of TC nanoemulsion decreased the tensile strength and the percentage of elongation at breaking, and slightly increased YM. Pectin films had a TS of 3.03 MPa, which decreased to 1.61 MPa after nanoemulsion addition. The same reduction occurred for EB from 8.16% to 4.14%. These findings are in accordance with the work of Sanchez-Gonzalez et al. [35], who observed a reduction in TS of hydroxy propyl methyl cellulose (HPMC) and chitosan films incorporated with bergamot, lemon, and tea tree essential oils. Similarly, chitosan-based films containing different concentrations of bergamot essential oil showed a significant decrease in the mechanical parameters. TS and YM decreased more than 50% and the EB was also considerably reduced from 22% to 5% compared to the films of chitosan alone. This reduction could be ascribed to the structural discontinuities in the polymer network of polysaccharide-based films, caused by the incorporation of the lipid-dispersed phase. However, some other authors obtained conflicting results. Indeed, an increase in mechanical properties of pectin films (TS, EB and YM) through the incorporation of clove bud essential oils was reported by Nisar et al. [15] similar to the results reported by Ghanbarzadeh et al. [33].

The EC film samples demonstrated better mechanical properties compared with monolayer films of P and PNE with a TS of 13.69 MPa and an EB of 7.52%. They also have considerably higher YM values compared to the other films, which could be due to the stiff nature of EC.

Multilayer films had a significantly higher TS compared to pectin and PNE films, which are around 14.98 MPa. However, the TS of multilayer films was slightly higher than that of EC monolayer films, which could be attributed to the effect of combining the EC layer with the PNE film which has a low tensile strength. Indeed, multilayer films preferably combined the mechanical properties of the EC film and those of the PNE film. This result shows better ductility for the multilayer samples. In addition, the film resistance to break was greatly improved by making three layers, which is higher than all three monolayer samples of P, PNE and EC. The Young’s modulus of multilayer films was lower than that of the EC monolayer, which presents a lower stiffness for the multilayer compared to EC, but it is still higher than the YM of P and PNE samples. On the other hand, the cross-sectional area of the film increases with thickness, and therefore, YM decreases. Therefore, the YM of thicker multilayer samples was lower than the YM of the EC monolayer. At the same time, a stiffer material will have a higher elastic modulus. The stiffness of the multilayer film is reduced compared to the EC monolayer film, which could be attributed to the pectin interlayer containing essential oils.

The obtained results are in agreement with recent studies that developed multilayer films based on biopolymers. Martucci et al. [36] reported an increase in TS and EM values of gelatin-based three-layer films. In addition, Xia et al. [37] fabricated three-layer films based on gelatin and zein. They achieved favorable flexibility and higher deformation resistance for the multilayer gelatin-zein films depending on the zein/gelatin ratio. Finally, it is important to note that during the tensile testing process, no layer separation was observed in all multilayer films, indicating strong adhesion between the layers.

### 2.7. Morphological Properties of Films

**Figure 1:**
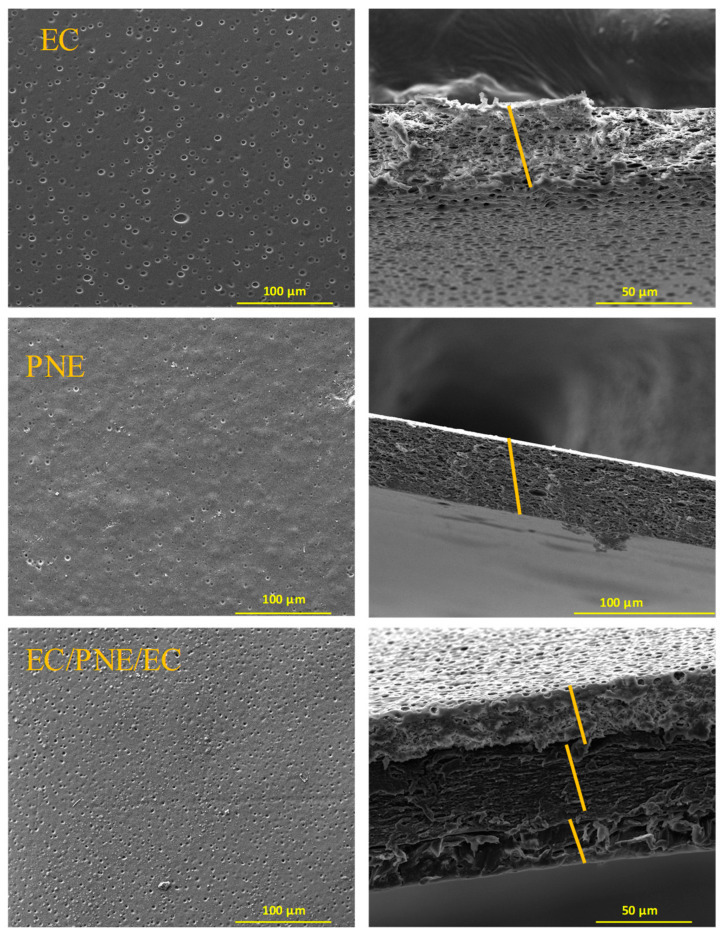
Surface (**left**) and cross-sectional (**right**) structures of monolayer films (EC and PNE) and multilayer films (EC/PNE/EC).

The morphological analysis was applied to evaluate the surface morphology and cross-sectional structure of the films. The SEM images are shown in Figure 1. A smooth surface morphology with some pores was observed for the PNE film. The cross-sectional images revealed a homogeneous and rather firm structure of the PNE film. The EC film also exhibited a smooth surface with the presence of some pores, more than the PNE film. The cross-sectional images represented an amorphous structure for EC films. Some porosity was observed on the surface of all samples which could be due to the fast evaporation rate of the solvent during the drying process. The surface morphology of multilayer films was similar to that of EC films. Three distinct layers could be clearly observed with a good adhesion in the cross-sectional image of multilayer films.

### 2.8. Antimicrobial Analysis

#### 2.8.1. In Vitro Antimicrobial Activity of Films

**Figure 2:**
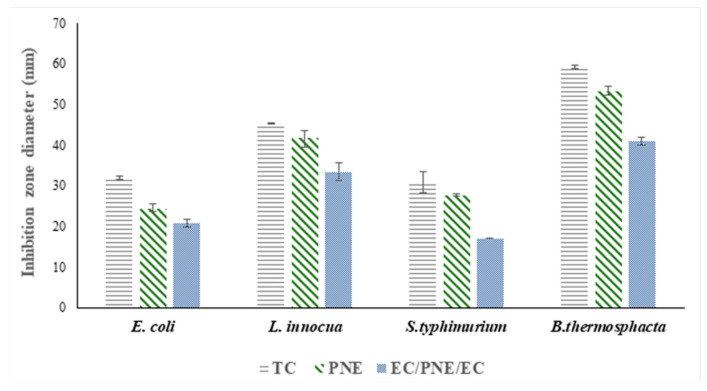
Inhibition zone diameters include clear zone diameter and 16 mm diameter film disc (PNE or EC/PNE/EC) or 8 mm diameter well (TC). TC: *trans*-Cinnamaldehyde, PNE: pectin film incorporated by nanocapsules of *trans*-Cinnamaldehyde, EC/PNE/EC: multilayer of PNE isolated by two outer layers of ethyl cellulose.

Among the tested samples, pectin monolayer film containing nanoemulsions of *trans*-Cinnamaldehyde (PNE) and multilayer samples (EC/PNE/EC) demonstrated antimicrobial activities against *L. innocua*, *E. coli*, *S. Typhimurium* and *Brochothrix thermosphacta* bacteria. The diameters of inhibition zones are shown in Figure 2.

As expected, the pectin and ethyl cellulose monolayers without nanoemulsions of *trans*-Cinnamaldehyde showed no inhibition zones, indicating that they have no antimicrobial activity against the tested bacteria, therefore they are not mentioned in Figure 2. However, the *trans*-Cinnamaldehyde (TC) oils showed effective antimicrobial activity against all four tested bacteria. As the EOs have a high potential to interact with the bacterial cell membrane and disrupt the cell configuration, they will induce ion exchange, leakage, permeability alteration, and respiration inhibition, leading to bacterial death [38].

In this study, the antimicrobial activity of TC was investigated not only to evaluate its antimicrobial activity but also to explore the capability of active films to release the emulsified TC on the surface of contaminated agar plates. The inhibition zones for all four bacteria showed the same trend. The largest diameter of the inhibition zones for all bacteria was observed for TC oil, as it acted directly in the inhibiting bacterial growth. Secondly, the PNE film samples showed larger zones of inhibition than multilayer samples (EC/PNE/EC). This result could be attributed to the effect on the EC layer, which slows down the release of TC molecules. The diameters of the inhibition zones were statistically different (*p <* 0.05) between the bacteria and followed the increasing order of *S. Typhimurium* < *E. coli* < *L. innocua* < *Brochothrix thermosphacta*. Among the four tested bacteria, the two Gram-positive bacteria *Brochothrix thermosphacta* and *L. innocua* demonstrated the larger inhibition zone diameters compared to Gram-negative bacteria *S.Typhimurium* and *E. coli.* This may be due to the possession of an additional hydrophilic membrane embedded with lipopolysaccharide molecules in Gram-negative bacteria that impedes the penetration of hydrophobic compounds such as essential oils. The absence of an additional lipophobic outer membrane in Gram-positive bacteria allows for an easier penetration of active compounds with greater inhibitory activity [39]. High antifungal and antimicrobial efficacy was reported for poly(lactic acid) (PLA) and poly(butylene adipate co terephthalate) (PBAT) blends incorporated with *trans*-Cinnamaldehyde according to Srisa et al. [40].

#### 2.8.2. Antimicrobial Activity of Mono and Multilayer Active Films on Refrigerated Stored Ground Beef Patties

**Figure 3:**
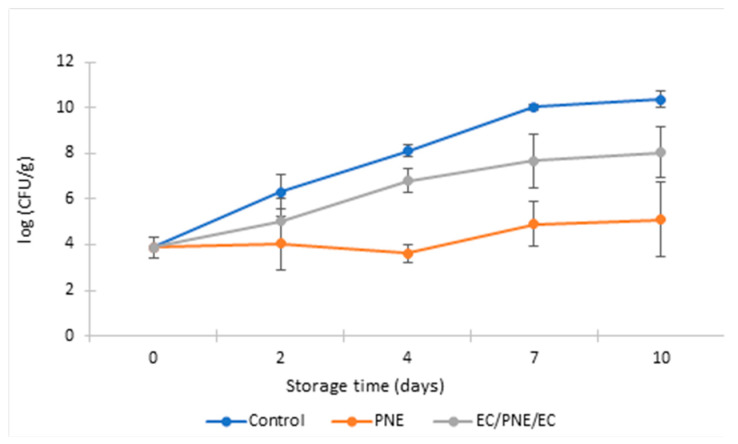
TVC of refrigerated stored ground beef patties packed with monolayer and multilayer active films during storage at 8 °C (log_10_ (CFU.g^−1^)).

Microbial growth of packed and unpacked ground beef patties was studied during 10 days of storage at 8 °C with sampling at days 0, 2, 4, 7 and 10. Results for total viable count (TVC) are presented in Figure 3. Study of TVC that grow aerobically at 30 °C is a global indicator for sanitary quality. At the beginning of storage, at day 0, all samples had the same value of TVC (3.87 ± 0.47 log_10_ CFU.g^−1^). However, during storage, the antimicrobial activity of the film samples decreased the total viable bacteria for both samples packed with pectin monolayer-containing nanoemulsion (PNE) and multilayer film with two outer layers of ethyl cellulose and a middle layer of pectin-containing nanoemulsion (EC/PNE/EC). Total bacterial growth declined for all packed samples compared with non-packed samples during the entire storage period. However, PNE films showed a higher efficiency in slowing down the bacterial growth compared to the samples packed with multilayer films. The main reason for this phenomenon is the solubility of pectin in water, which causes the PNE film to adhere to the patty samples. It is practically impossible to separate ground beef patties from the film in which they were wrapped. Therefore, all amounts of *trans*-Cinnamaldehyde as antimicrobial agent were mixed with the ground beef patty samples during the test. As a result, the active pectin films showed a better efficiency in inhibiting bacterial growth. At the same time, the solubility of pectin film in water limits its application as an active film for the preservation of foodstuffs with a high water content.

The TVC counted for samples packed with PNE film was 5.10 log_10_ CFU.g^−1^ on the 10th day, which is significantly lower than the TVC of unpacked control samples with 10.30 log_10_ CFU.g^−1^ at the same day. On the other hand, the multilayer films protected the middle layer of pectin-containing nanoemulsion from solubilization. They also slowed down the total bacterial growth in the ground beef patties packed with it. Samples packaged with multilayer films had fewer total viable bacteria than non-packed control samples during all days of the storage period. This indicates the ability of this multilayer active film to delay food spoilage through the presence of nanoemulsions of TC on the food surface. The TVC of the unpacked control samples increased from 3.87 to 10.35 log_10_ (CFU.g^−1^) during the 10 days of storage. However, the TVC of patty samples packed with active multilayer samples was 8.0 log_10_ (CFU.g^−1^) at the end of the storage period. In addition, the shape and texture of EC/PNE/EC film samples after packaging did not change and film samples remained intact.

**Figure 4:**
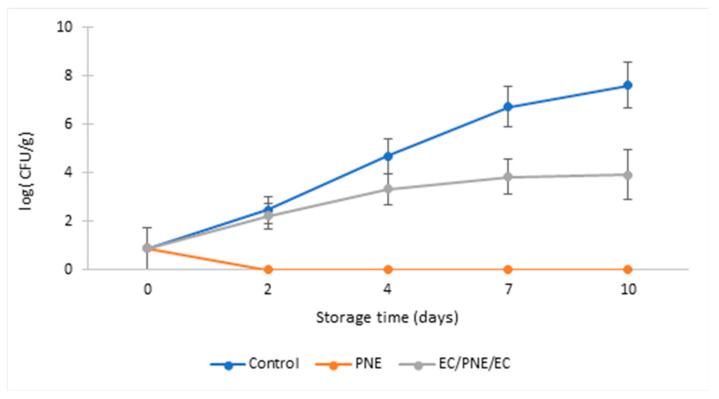
TCC of refrigerated stored ground beef patties packed with monolayer and multilayer active films during storage at 8 °C (log_10_ (CFU.g^−1^)).

Total coliform count (TCC) was carried out to investigate the capacity of this active packaging film to inhibit or slow down coliform growth on ground beef patties during refrigerated storage. The coliforms, which regroup numerous species such as *E. coli*, are Gram-negative bacteria that ferment lactose and belong to the family *Enterobacteriaceae*. The results are given in Figure 4. Both monolayer and multilayer sample films showed lower TCC than the control samples. This indicates the effectiveness of these packaging films to inhibit or retard coliform bacteria growth. For the same reason mentioned above, the ground beef patty samples packed with PNE films showed lower total coliform growth than the control sample and the samples packed with EC/PNE/EC films. The TCC at the beginning of storage at 8 °C was 0.90 log_10_ CFU.g^−1^; at the end of the refrigerated storage (10 days at 8 °C), TCC was 7.62 log_10_ CFU.g^−1^ for the control samples and 3.92 log_10_ CFU.g^−1^ for the samples packed with EC/PNE/EC film. PNE film samples completely inhibited coliform growth during refrigerated storage.

**Figure 5:**
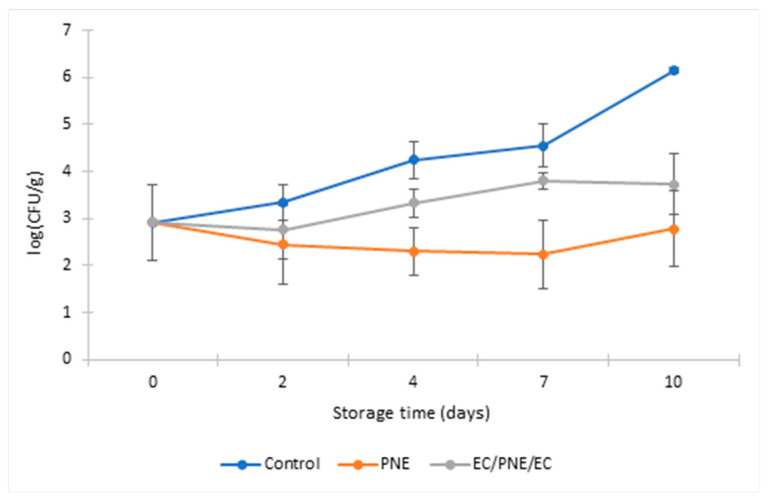
Count of lactic acid bacteria (LAB) of refrigerated stored ground beef patties packed with monolayer and multilayer active films during storage at 8 °C (log_10_ (CFU.g^−1^)).

Lactic Acid Bacteria (LAB) are important spoilage organisms in cold-stored meat products. The effect of the obtained active packaging on the growth of lactic acid bacteria (LAB) was also investigated during the storage of ground beef patties packed with different films at 8 °C, and the results are presented in Figure 5. Samples packed with active monolayer and multilayer films exhibited less growth of lactic acid bacteria compared to unpacked samples. The LAB at the beginning of storage at 8 °C was 2.92 log_10_ CFU.g^−1^; at the end of the refrigerated storage (10 days at 8 °C), LAB was 6.15 log_10_ CFU.g^−1^ for control samples, 1.85 log_10_ CFU.g^−1^ for patty samples packed with PNE film, and 3.73 log_10_ CFU.g^−1^ for patty samples packed with EC/PNE/EC film, respectively. Numerous studies have been conducted on the active packaging of ground beef or meat patties using different packaging materials and bioactive substances. Ground beef patties packed with edible zein films incorporating partially purified lysozyme and Na_2_EDTA showed a significant decrease in total viable counts (TVC) and total coliform counts after 5 days of storage compared with unpacked control patties [41]. In a recent study, the essential oil of *Cinnamodendron dinisii* Schwanke was nanoencapsulated by zein as wall material, and incorporated into the chitosan matrix. The active chitosan film containing Cinnamodendron Dinisii essential oil nanoparticles showed its ability to stabilize spoilage reactions of ground beef under refrigerated storage and increased the shelf life of the samples [42]. An active film of cassava starch incorporated with oregano essential oil and pumpkin residue extract also showed antimicrobial activity against mesophilic bacteria, coliforms, and *Salmonella* on ground beef according to Caetano et al. [43]. In another research work, active packaging including an inner surface coated with a chitosan film with different concentrations of thyme essential oil concentrations was used for packaging, but not in direct contact with meat. The color change, microbial populations, and sensory properties were assessed during 4 weeks of refrigerated storage. Results showed a reduction in yeast populations and preservation of meat color as well as water condensation inside the package [44].

## 3. Materials and Methods

### 3.1. Materials

Ethylcellulose powder 48.0–49.5% (*w/w*) ethoxyl basis was purchased from Sigma Aldrich (Darmstadt, Germany), and *trans*-Cinnamaldehyde (TC, 99%) was obtained from Sigma-Aldrich (St Quentin Fallavier, France). Granular soybean Lecithin was obtained from Acros Organics (Geel, Belgium). Low methoxyl pectin (LMP, esterification degree from 22% to 28% and acetylation degree from 20% to 23%) with 81.21% carbohydrate content was bought from Cargill (Baupte, France). Glycerol used as plasticizer was purchased from AppliChem GmbH (Darmstadt, Germany).

### 3.2. Preparation of the Nanoemulsions (NE)

Five percent (*w/w*) of TC was dispersed in distilled water by emulsifying 1% (*w/w*) of soybean lecithin using a high-speed homogenizer (POLYTRON PT 2500 E, KINEMATIC, Swiss) for 6 min under 15,000 rpm to form coarse emulsion. Finally, nanoemulsion was obtained by passing coarse emulsion through a microfluidizer (Microfluidizer LM20, Microfluidics Corp, Newton, MA, USA) two times at a pressure of 500 bar and one time at a pressure of 1000 bar.

### 3.3. Preparation of Active Multilayer Films

#### 3.3.1. Preparation of Film-Forming Solutions

Six percent (*w/w*) ethyl cellulose and 1% (*w/w*) glycerol as plasticizer were solubilized in ethanol absolute by stirring on the magnetic stirrer for 4 h for the preparation of the ethyl cellulose film-forming solution (EC). Three percent (*w/w*) pectin and one percent (*w/w*) glycerol were solubilized in distilled water by stirring on the magnetic stirrer for 2 h at room temperature for the preparation of the pectin solution (P). Then, for the preparation of the active film-forming solution (PNE), the pectin solution was mixed with NE at ratio of 1:1 for about 1 h by stirring at ambient temperature.

#### 3.3.2. Preparation of Films

Different films were prepared with different compositions, as shown in Table 3.

**Figure 6:**
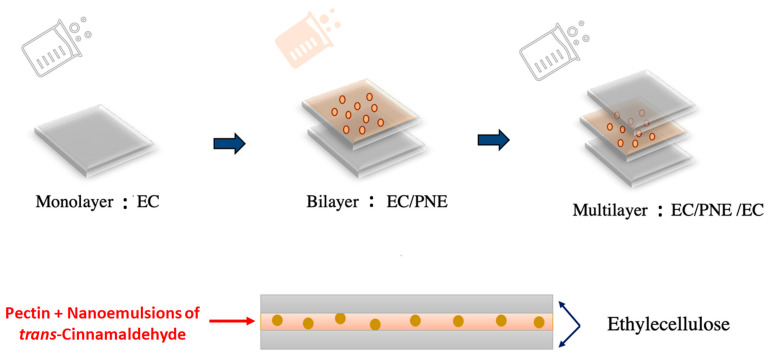
Fabrication of active multilayer films (EC/PNE/EC).

Monolayer films were prepared by pouring film solutions into glass Petri dishes, which were then dried in an oven at 60 °C for 2, 4 and 6 h for monolayer films of EC, P, or PNE, respectively. Multilayer active films were also fabricated by casting the film-forming solutions into glass Petri dishes, followed by drying at 60 °C in an oven according to the layer-by-layer method. The first layer of EC was cast and dried for 2 h; then, the second layer of PNE solution was added and kept overnight until dry; and finally, the last layer of EC was poured into and dried for 5 h (Figure 6).

### 3.4. Characterization of Nanoemulsions

#### 3.4.1. Size Distribution

Droplet size and size distribution of nanoemulsions were determined by dynamic light scattering (DLS) using a Ζetasizer Nano-ZS90 (Malvern Instruments Ltd., Worcestershire, UK) at a fixed angle of 90°. One mL of nanoemulsion was diluted and homogenized into ten mL of distilled water at room temperature (23 ± 1 °C) to eliminate multiple scattering effects. After 90 s of equilibrium, the measurement was performed. The measurements were repeated three times and the average droplet size and average polydispersity index (PDI) were calculated.

#### 3.4.2. Zeta Potential (ζ)

The zeta potential of nanoemulsion particles was measured with a Ζetasizer Nano-ZS90 (Malvern Instruments Ltd., Worcestershire, UK) in disposable cuvettes for an electrophoretic mobility measurement. The samples were prepared by diluting 1 mL of nanoemulsion into 10 mL of distilled water and homogenizing completely. The zeta potential measurement was fulfilled in triplicate and the mean value and standard deviation were calculated.

### 3.5. Physicochemical Characterization of Films

#### 3.5.1. Film Thickness

The thickness of film samples was determined using an electronic micrometer (WLMART 0–25 mm, 0.001 mm). Measurements were performed at more than three different points of each film surface to calculate the average values.

#### 3.5.2. Light Transmission and Transparency of Films

The light barrier properties of films against visible light and ultraviolet (UV) were determined according to the method of Peng et al. [45]. Rectangular film strips (9 mm × 40 mm) were placed in the spectrophotometer cuvette and the absorbance was measured at 600 nm by a spectrophotometer UV-3100PC, VWR (Rosny-sous-Bois, France). The opacity of the films was calculated using Equation (1):

(1) $$O=\frac{Abs\;600}{L}$$

where *O* (A.mm^−1^) is the opacity of films, *Abs* 600 is the value of absorbance at 600 nm (A), and *L* is the film specimen thickness (mm).

#### 3.5.3. Moisture Absorption

Moisture absorption of the film samples was measured by the method of Ghadetaj et al. [46] with some modifications. Firstly, film specimens of 20 mm × 20 mm squares were conditioned at 0% relative humidity (RH) into a desiccator containing silica gel for 3 days and weighed (*W*0). The samples were then placed in a desiccator containing saturated NaCl solution to condition them to an RH of 75% at room temperature. The samples were weighed periodically with a precision of 0.001 mg until a constant weight was reached (*Wt*). The moisture absorption percentage was calculated using Equation (2).

(2) $$Moisture\;absorption\;(\%)=\frac{W0-Wt}{W0}\times 100$$

#### 3.5.4. Mechanical Properties

The mechanical parameters, including Young’s modulus (YM), tensile strength (TS), and elongation at break (%EB) of the film samples were investigated by a tensile analyzer (EZ Test EZ-X Series, Shimadzu Corporation, Kyoto, Japan) with a 500 N load cell connected to an autograph software (Trapezium X, Shimadzu Corporation, Japan). The tests were carried out following the ASTM D882 standard method. The film samples were cut into a square shape of 15 mm × 100 mm. After conditioning at an RH of 54% for one week (saturated solution of Mg(NO_3_)_2_), they were mounted in two grips with a specimen’s gauge length of 50 mm; then, the tests were performed at a crosshead speed of 10 mm/min. Mechanical parameters were calculated by the computer-recorded force-deformation curves with the following equations:

(3) $$TS=\frac{Force\;at\;break}{Film\;section}$$

(4) $$EB\;(\%)=\frac{Change\;in\;length}{Initial\;film\;length}\times 100$$

(5) $$YM=\frac{curve\;slope}{Film\;section}\times Initial\;film\;length$$

where the film section is width × thickness.

#### 3.5.5. Scanning Electron Microscopy (SEM)

The morphological analysis was performed using Scanning Electron Microscopy (FEI Quanta 250 microscope (Eindhoven, The Netherland)) at the “Centre Technologique des Microstructures” (CTμ) at the University of Claude Bernard Lyon 1 (Villeurbanne, France). Film samples were deposited on a flat steel holder. The samples were coated under a vacuum by cathodic sputter before performing microscopy analysis.

### 3.6. Antimicrobial Activity of Films

#### 3.6.1. In vitro Antimicrobial Activity

The antibacterial effect of samples was determined by using the disk diffusion method against two Gram-positive bacteria, *Listeria innocua* (DSM20649) and *Brochothrix thermosphacta* (DSM20171), and two Gram-negative bacteria, *Escherichia coli* (DSM613) and *Salmonella typhimurium* (DSM11320). The strains were provided by DSMZ-German Collection of Microorganisms. Strains were stored at −20 °C in Tryptone Soy Broth (TSB) (Biokar diagnostics, Beauvais, France) with 15% (*v/v*) of glycerol. One milliliter of the stock culture was transferred to nine milliliters of TSB and incubated overnight at 30 °C (*B. thermosphacta*) or 37 °C (*E. coli*, *L. innocua* and *S. typhimurium*). One milliliter of this pre-culture was transferred in nine milliliters of TSB and incubated for 5 h at 30 ± 1 °C or 37 ± 1 °C. Finally, 10^6^ CFU.mL^−1^ of each bacterial strain was spread on the surface of Tryptone Soy Agar plates (TSA) (Biokar diagnostics, Beauvais, France). EC/PNE/EC multilayer films were cut in 16 mm-diameter discs with a hole puncher and placed on inoculated plates. Monolayers of pectin (P) and ethylcallulose (EC) film samples were tested as controls. In addition, the antimicrobial activity of free *trans*-Cinnamaldehyde was investigated as a reference for comparison with encapsulated *trans*-Cinnamaldehyde incorporated into the films. A well with a diameter of 8 mm was punched aseptically in the contaminated agar plate. As each 16 mm disc of active film contained about 86 μL of *trans*-cinnamaldehyde, 86 µL of *trans*-Cinnamaldehyde was introduced into the well. The agar plates inoculated with *Brochothrix thermosphacta* were incubated at 30 ± 1 °C and the plates inoculated with the other strains were incubated at 37 ± 1 °C for 24 h. The diameters of the inhibition zones around the film discs and around the wells were measured. The experiments were made in triplicate for each film.

#### 3.6.2. Efficiency of the Prepared Active Films during the Storage of Packed Ground Beef

Ground beef was obtained from a local butcher shop. It was hand mixed aseptically using gloves for 1 min to be completely homogenized. This ground beef mixture was then divided into portions of 10 g and 3 types of samples: (1) control; (2) samples coated with active pectin films PNE; and (3) samples coated with active multilayer films EC/PNE/EC. The samples were prepared in sterile plastic Petri dishes of 60 mm. Unpacked samples were used as controls. Samples 2 and 3 were packed with putting films (PNE or EC/PNE/EC) on the upper and bottom surface of the ground beef. All samples were stored at 8 °C for 10 days for sampling at days 0, 2, 4, 7 and 10 for further analysis. The films were peeled off from sample surfaces after each day of analysis. Samples were homogenized in sterile stomacher bags with 90 mL of sterile tryptone salt broth (Biokar diagnostics, Beauvais, France) using a stomacher (Imix, Interlab, Germany) for 90 s. Serial decimal dilutions were made in tryptone salt (Biokar diagnostics, Beauvais, France) until the appropriate dilutions were reached. One mL of each selected dilution was poured into petri dishes, culture media was added, and then petri dishes were incubated at the appropriate temperature. Total Viable Counts (TVC) were enumerated on TSA (Biokar diagnostics, Beauvais, France) incubated at 30 °C for 72 h. Lactic acid bacteria were enumerated on De Man, Rogosa and Sharp agar (MRS) (Biokar diagnostics, Beauvais, France) and incubated for 72 h at 37 °C. Total coliforms were enumerated on Violet Red Bile Lactose agar (VRBL) (Biokar diagnostics, Beauvais, France) and incubated for 24 h at 37 °C. Experiments were performed in triplicate. The microbial counts were expressed as log10 of colony-forming units (CFU) per gram of sample.

### 3.7. Statistical Analysis

All tests were performed at least in triplicate. The data were presented as means ± standard deviation for different samples. One-way analysis of variance (ANOVA) was used, followed by Fisher’s test (F) to compare the means. Difference was considered significant at *p <* 0.05.

## 4. Conclusions

In this work, biodegradable antimicrobial active film packaging was designed via a layer-by-layer solvent-casting method. A comparison between a monolayer film of pectin incorporating *trans*-Cinnamaldehyde nanoemulsion and a multilayer film containing the active pectin monolayer isolated by the inner and outer layer of ethyl cellulose was performed. In particular, the physical and mechanical properties of monolayer films were improved by the multilayer technique. Antimicrobial activities on four target bacteria and ground beef patties demonstrated the antimicrobial activity for both mono and multilayer active films. At the same time, the multilayer technique modified the water sensitivity problem of monolayer pectin films. Hydrophobic layers of ethyl cellulose protect active pectin layers in contact with humid foodstuffs. Therefore, this multilayer film packaging design could be useful to extend the shelf life of fresh and perishable foods.

## Figures and Tables

**Table 1:** Physical properties of mono and multilayer films.

Film Samples	Thickness (μm)	Opacity (A × mm^−1^)	Moisture Absorption (%)
P	44.33 ± 2.08 ^c^	1.20 ± 0.02 ^b^	22.54 ± 1.81 ^a^
PNE	62.00 ± 2.65 ^c^	15.19 ± 1.55 ^a^	9.40 ± 2.28 ^c^
EC	105.33 ± 7.02 ^b^	0.97 ± 0.06 ^b^	7.21 ± 0.20 ^c^
EC/PNE/EC	285.67 ± 18.65 ^a^	2.02 ± 0.14 ^b^	15.35 ± 0.64 ^b^

The data represent the mean ± one standard deviation (n = 3). Means with the same letter are not significantly different (*p* < 0.05).

**Table 2:** Mechanical properties of mono and multilayer films.

Symbol of Samples	Tensile Strength (MPa)	Elongation at Break (%)	Young Module (MPa)
P	3.03 ± 0.27 ^b^	8.16 ± 1.08 ^ab^	42.18 ± 3.10 ^c^
PNE	1.61 ± 0.28 ^b^	4.14 ± 1.43 ^c^	48.15 ± 11.69 ^c^
EC	13.69 ± 1.38 ^a^	7.52 ± 0.76 ^b^	220.37 ± 24.14 ^a^
EC/PNE/EC	14.98 ± 0.90 ^a^	10.20 ± 0.98 ^a^	179.22 ± 23.02 ^b^

The data represent the mean ± one standard deviation (n = 3). Means with the same letters are not significantly different (*p* < 0.05).

**Table 3:** Composition of films.

Sample Names	Composition of the Film
P	Monolayer of Pectin
PNE	Monolayer of pectin incorporated by nanoemulsion of *trans*-Cinnamaldehyde
EC	Monolayer of ethyl cellulose
EC/PNE/EC	Multilayer including two outer layers of EC and one intermediate layer of PNE

## Data Availability

Not applicable.

## Sample Availability

Samples of the compounds not available from the authors.

## References

[1] Walther B.A., Kusui T., Yen N., Hu C.-S., Lee H., Stock F., Reifferscheid G., Brennholt N., Kostianaia E. (2022). Plastic Pollution in East Asia: Macroplastics and Microplastics in the Aquatic Environment and Mitigation Efforts by Various Actors. Plastics in the Aquatic Environment—Part I: Current Status and Challenges.

[2] Defruyt S. (2019). Towards a New Plastics Economy. Field Actions Science Reports.

[3] Fabra M.J., López-Rubio A., Lagaron J.M., Aguilar M.R., San Román J. (2014). 15—Biopolymers for Food Packaging Applications. Smart Polymers and their Applications.

[4] Luo S., Chen R., Huang L., Liang R., Liu C., Chen J. (2017). Investigation on the Influence of Pectin Structures on the Pasting Properties of Rice Starch by Multiple Regression. Food Hydrocoll..

[5] Gao H.-X., He Z., Sun Q., He Q., Zeng W.-C. (2019). A Functional Polysaccharide Film Forming by Pectin, Chitosan, and Tea Polyphenols. Carbohydr. Polym..

[6] Dranca F., Oroian M. (2018). Extraction, Purification and Characterization of Pectin from Alternative Sources with Potential Technological Applications. Food Res. Int..

[7] Espitia P.J.P., Du W.-X., de Avena-Bustillos R.J., de Soares N.F.F., McHugh T.H. (2014). Edible Films from Pectin: Physical-Mechanical and Antimicrobial Properties—A Review. Food Hydrocoll..

[8] Quinto E.J., Caro I., Villalobos-Delgado L.H., Mateo J., De-Mateo-Silleras B., Redondo-Del-Río M.P. (2019). Food Safety through Natural Antimicrobials. Antibiotics.

[9] Burt S. (2004). Essential Oils: Their Antibacterial Properties and Potential Applications in Foods—A Review. Int. J. Food Microbiol..

[10] Aljaafari M.N., AlAli A.O., Baqais L., Alqubaisy M., AlAli M., Molouki A., Ong-Abdullah J., Abushelaibi A., Lai K.-S., Lim S.-H.E. (2021). An Overview of the Potential Therapeutic Applications of Essential Oils. Molecules.

[11] Chen H., Hu X., Chen E., Wu S., McClements D.J., Liu S., Li B., Li Y. (2016). Preparation, Characterization, and Properties of Chitosan Films with Cinnamaldehyde Nanoemulsions. Food Hydrocoll..

[12] Mukurumbira A.R., Shellie R.A., Keast R., Palombo E.A., Jadhav S.R. (2022). Encapsulation of Essential Oils and Their Application in Antimicrobial Active Packaging. Food Control.

[13] Jafari S.M., Jafari S.M. (2017). 1—An Overview of Nanoencapsulation Techniques and Their Classification. Nanoencapsulation Technologies for the Food and Nutraceutical Industries.

[14] Sharma R., Jafari S.M., Sharma S. (2020). Antimicrobial Bio-Nanocomposites and Their Potential Applications in Food Packaging. Food Control.

[15] Nisar T., Wang Z.-C., Yang X., Tian Y., Iqbal M., Guo Y. (2018). Characterization of Citrus Pectin Films Integrated with Clove Bud Essential Oil: Physical, Thermal, Barrier, Antioxidant and Antibacterial Properties. Int. J. Biol. Macromol..

[16] Almasi H., Azizi S., Amjadi S. (2020). Development and Characterization of Pectin Films Activated by Nanoemulsion and Pickering Emulsion Stabilized Marjoram (*Origanum majorana* L.) Essential Oil. Food Hydrocoll..

[17] Alvarez M.V., Ortega-Ramirez L.A., Gutierrez-Pacheco M.M., Bernal-Mercado A.T., Rodriguez-Garcia I., Gonzalez-Aguilar G.A., Ponce A., del Moreira M.R., Roura S.I., Ayala-Zavala J.F. (2014). Oregano Essential Oil-Pectin Edible Films as Anti-Quorum Sensing and Food Antimicrobial Agents. Front. Microbiol..

[18] Guerra-Rosas M.I., Morales-Castro J., Cubero-Márquez M.A., Salvia-Trujillo L., Martín-Belloso O. (2017). Antimicrobial Activity of Nanoemulsions Containing Essential Oils and High Methoxyl Pectin during Long-Term Storage. Food Control.

[19] Yang D., Peng X., Zhong L., Cao X., Chen W., Zhang X., Liu S., Sun R. (2014). “Green” Films from Renewable Resources: Properties of Epoxidized Soybean Oil Plasticized Ethyl Cellulose Films. Carbohydr. Polym..

[20] Ju J., Chen X., Xie Y., Yu H., Guo Y., Cheng Y., Qian H., Yao W. (2019). Application of Essential Oil as a Sustained Release Preparation in Food Packaging. Trends Food Sci. Technol..

[21] Wang Q., Chen W., Zhu W., McClements D.J., Liu X., Liu F. (2022). A Review of Multilayer and Composite Films and Coatings for Active Biodegradable Packaging. Npj Sci. Food.

[22] Almasi H., Jahanbakhsh Oskouie M., Saleh A. (2021). A Review on Techniques Utilized for Design of Controlled Release Food Active Packaging. Crit. Rev. Food Sci. Nutr..

[23] Mastromatteo M., Barbuzzi G., Conte A., Del Nobile M.A. (2009). Controlled Release of Thymol from Zein Based Film. Innov. Food Sci. Emerg. Technol..

[24] Stroescu M., Stoica-Guzun A., Jipa I.M. (2013). Vanillin Release from Poly(Vinyl Alcohol)-Bacterial Cellulose Mono and Multilayer Films. J. Food Eng..

[25] Buonocore G.G., Conte A., Corbo M.R., Sinigaglia M., Del Nobile M.A. (2005). Mono- and Multilayer Active Films Containing Lysozyme as Antimicrobial Agent. Innov. Food Sci. Emerg. Technol..

[26] Jipa I.M., Stoica-Guzun A., Stroescu M. (2012). Controlled Release of Sorbic Acid from Bacterial Cellulose Based Mono and Multilayer Antimicrobial Films. LWT.

[27] Xiong Y., Li S., Warner R.D., Fang Z. (2020). Effect of Oregano Essential Oil and Resveratrol Nanoemulsion Loaded Pectin Edible Coating on the Preservation of Pork Loin in Modified Atmosphere Packaging. Food Control.

[28] Bogdanović U., Lazić V., Vodnik V., Budimir M., Marković Z., Dimitrijević S. (2014). Copper Nanoparticles with High Antimicrobial Activity. Mater. Lett..

[29] Mudalige T., Qu H., Van Haute D., Ansar S.M., Paredes A., Ingle T., López Rubio A., Fabra Rovira M.J., Martínez Sanz M., Gómez-Mascaraque L.G. (2019). Chapter 11—Characterization of Nanomaterials: Tools and Challenges. Nanomaterials for Food Applications.

[30] Duffy J., Larsson M., Hill A. (2012). Suspension Stability; Why Particle Size, Zeta Potential and Rheology Are Important. Annu. Trans. Nord. Rheol. Soc..

[31] Galdeano M.C., Wilhelm A.E., Mali S., Grossmann M.V.E. (2013). Influence of Thickness on Properties of Plasticized Oat Starch Films. Braz. Arch. Biol. Technol..

[32] Escamilla-García M., Reyes-Basurto A., García-Almendárez B.E., Hernández-Hernández E., Calderón-Domínguez G., Rossi-Márquez G., Regalado-González C. (2017). Modified Starch-Chitosan Edible Films: Physicochemical and Mechanical Characterization. Coatings.

[33] Ghanbarzadeh B., Almasi H. (2011). Physical Properties of Edible Emulsified Films Based on Carboxymethyl Cellulose and Oleic Acid. Int. J. Biol. Macromol..

[34] Fasihi H., Fazilati M., Hashemi M., Noshirvani N. (2017). Novel Carboxymethyl Cellulose-Polyvinyl Alcohol Blend Films Stabilized by Pickering Emulsion Incorporation Method. Carbohydr. Polym..

[35] Sánchez-González L., Chiralt A., González-Martínez C., Cháfer M. (2011). Effect of Essential Oils on Properties of Film Forming Emulsions and Films Based on Hydroxypropylmethylcellulose and Chitosan. J. Food Eng..

[36] Martucci J.F., Ruseckaite R.A. (2009). Biodegradation of Three-Layer Laminate Films Based on Gelatin under Indoor Soil Conditions. Polym. Degrad. Stab..

[37] Xia C., Wang W., Wang L., Liu H., Xiao J. (2019). Multilayer Zein/Gelatin Films with Tunable Water Barrier Property and Prolonged Antioxidant Activity. Food Packag. Shelf Life.

[38] Walsh S.E., Maillard J.-Y., Russell A.D., Catrenich C.E., Charbonneau D.L., Bartolo R.G. (2003). Activity and Mechanisms of Action of Selected Biocidal Agents on Gram-Positive and -Negative Bacteria. J. Appl. Microbiol..

[39] Fisher K., Phillips C.A. (2006). The Effect of Lemon, Orange and Bergamot Essential Oils and Their Components on the Survival of Campylobacter Jejuni, Escherichia Coli O157, Listeria Monocytogenes, Bacillus Cereus and Staphylococcus Aureus in Vitro and in Food Systems. J. Appl. Microbiol..

[40] Srisa A., Harnkarnsujarit N. (2020). Antifungal Films from Trans-Cinnamaldehyde Incorporated Poly(Lactic Acid) and Poly(Butylene Adipate-Co-Terephthalate) for Bread Packaging. Food Chem..

[41] Ünalan İ.U., Korel F., Yemenicioğlu A. (2011). Active Packaging of Ground Beef Patties by Edible Zein Films Incorporated with Partially Purified Lysozyme and Na2EDTA. Int. J. Food Sci. Technol..

[42] Xavier L.O., Sganzerla W.G., Rosa G.B., da Rosa C.G., Agostinetto L., de Veeck A.P.L., Bretanha L.C., Micke G.A., Dalla Costa M., Bertoldi F.C. (2021). Chitosan Packaging Functionalized with Cinnamodendron Dinisii Essential Oil Loaded Zein: A Proposal for Meat Conservation. Int. J. Biol. Macromol..

[43] dos Caetano K.S., Hessel C.T., Tondo E.C., Flôres S.H., Cladera-Olivera F. (2017). Application of Active Cassava Starch Films Incorporated with Oregano Essential Oil and Pumpkin Residue Extract on Ground Beef. J. Food Saf..

[44] Quesada J., Sendra E., Navarro C., Sayas-Barberá E. (2016). Antimicrobial Active Packaging Including Chitosan Films with *Thymus vulgaris* L. Essential Oil for Ready-to-Eat Meat. Foods.

[45] Peng Y., Li Y. (2014). Combined Effects of Two Kinds of Essential Oils on Physical, Mechanical and Structural Properties of Chitosan Films. Food Hydrocoll..

[46] Ghadetaj A., Almasi H., Mehryar L. (2018). Development and Characterization of Whey Protein Isolate Active Films Containing Nanoemulsions of Grammosciadium Ptrocarpum Bioss. Essential Oil. Food Packag. Shelf Life.

